# LncRNAs and CircRNAs as Strategies against Pathological Conditions Caused by a Hypoxic/Anoxic State

**DOI:** 10.3390/biom13111622

**Published:** 2023-11-06

**Authors:** Ivan Anchesi, Giovanni Schepici, Emanuela Mazzon

**Affiliations:** IRCCS Centro Neurolesi “Bonino-Pulejo”, Via Provinciale Palermo, Strada Statale 113, Contrada Casazza, 98124 Messina, Italy

**Keywords:** LncRNAs, CircRNAs, hypoxic brain damage, anoxic brain damage, HIBD (hypoxic ischemic brain damage)

## Abstract

Brain damage can be induced by oxygen deprivation. It is known that hypoxic or anoxic conditions can lead to changes in the expression levels of non-coding RNAs (ncRNAs), which, in turn, can be related to Central Nervous System (CNS) injuries. Therefore, it could be useful to investigate the involvement of non-coding RNAs (ncRNAs), as well as the underlying mechanisms which are able to modulate them in brain damage induced by hypoxic or anoxic conditions. In this review, we focused on recent research that associates these conditions with long non-coding RNAs (lncRNAs) and circular RNAs (circRNAs). The results of this review demonstrate that the expression of both lncRNAs and circRNAs can be influenced by oxygen deprivation conditions and so they can contribute to inducing damage or providing neuroprotection by affecting specific molecular pathways. Furthermore, several experimental studies have shown that ncRNA activity can be regulated by compounds, thus also modifying their transcriptomic profile and their effects on CNS damages induced by hypoxic/anoxic events.

## 1. Introduction

To maintain brain functions, appropriate oxygen delivery must be ensured to create enough energy via oxygen-dependent metabolic pathways. If the demands for oxygen are higher than the possible delivery, this leads to hypoxic brain damage, which can further continue to anoxic brain damage if the oxygen delivery is fully disrupted. Throughout life, there are numerous ways that hypoxic/anoxic conditions may occur, and this includes but is not limited to asphyxia [1], cardiac arrest [2], traumatic vascular injuries [3], septic shock [4], drug overdoses [5], carbon monoxide poisoning [6], severe hypotension [7], and brain cerebrovascular diseases such as ischemic stroke [8], as well as other anoxic/hypoxic conditions including obstructive sleep apnea-hypopnea syndrome (OSAHS) [9,10], heart failure [11,12], and acute lung injury [13].

It is known that non-coding RNAs (ncRNAs) play a role in neuronal development [14,15,16] and their expression is affected in pathological conditions or therapeutic contexts [17,18].

Despite advancements in genetics and proteomics demonstrating that some small-size ncRNAs contain short open reading frames able to encode small proteins involved in many biological processes [19], ncRNAs are primarily classified as RNAs that do not translate into proteins [20]. It has been reported that ncRNAs can act with nucleic acids as regulatory molecules both in physiological conditions and in several diseases, particularly those involving the central nervous system (CNS). Several ncRNAs, including microRNAs, long non-coding RNAs (lncRNAs), and circular RNAs (circRNAs), are dysregulated in CNS diseases [21,22,23].

NcRNAs are categorized into structural non-coding RNAs, such as ribosomal RNA and transfer RNA, and regulatory non-coding RNAs, including lncRNAs and circRNAs [22,24].

LncRNAs constitute a diverse group of RNAs that do not translate into proteins. Their biogenesis is similar to that of messenger RNAs, which are transcribed by polymerase II (Pol II), polyadenylated at the 3′ end, and capped at the 5′ end [25]. After transcription, lncRNAs fold into thermodynamically stable secondary structures [26].

Additionally, lncRNAs possess four functional domains, including RNA-binding domains, protein-binding domains, DNA-binding domains, and conformational switch [25,26]. By switching structural conformations and linking binding domains, they exert regulatory functions [25].

LncRNA are divided based on their structure (linear lncRNA, circular RNAs, long intervening/intergenic noncoding RNAs, enhancer RNAs, transcribed ultra-conserved RNAs, and natural antisense transcript), action (cis-acting long non-coding RNA, competing endogenous RNA, and trans-acting long non-coding RNA), and biogenesis [25]. Depending on their genome localization and biogenesis, they can be classified and further divided into subclasses [24] such as sense lncRNAs or antisense lncRNAs, intronic lncRNAs, and intergenic lncRNAs [27,28,29].

LncRNAs can regulate gene expression through several mechanisms, including the modulation of splicing machinery, transcription factor activity, or gene promoters. They also act as structural components in protein complex assembly and serve as competing endogenous RNAs (ceRNAs) to modulate miRNA functions [23,30]. In this regard, lncRNAs can inhibit or activate target genes, by directly binding to them or by recruiting transcription factors and thus modulating many cellular processes, including cell proliferation, differentiation, apoptosis, autophagy, angiogenesis, and immune response [31,32].

Furthermore, it has been demonstrated that lncRNAs can interact with microRNAs (miRNAs) to regulate target proteins, thus playing a significant role in molecular regulation, such as cell differentiation [33,34].

In this regard, alterations in the levels of lncRNAs have been reported in neural tissue after ischemia and hypoxia [35], especially in the early stages of the ischemic event [36].

Moreover, it was observed that lncRNAs related to hypoxic conditions play a regulatory role in inflammatory events and oxidative stress, thus contributing to the onset and progression of ischemic events [37].

In this review, we focused also on circular RNAs (circRNAs), a type of non-coding RNA that forms a covalently closed loop structure [23,38].

CircRNAs include exon intron circRNAs (EIciRNAs), circular intron RNAs (ciRNAs), and exon circRNAs (ecircRNAs). Although most circRNAs are found in the cytoplasm, ciRNAs and EIciRNAs were found in the nucleus of human cells [39,40].

Functionally, one of the main features of circRNAs is the ability of binding with complementary miRNAs, acting as so called “miRNA sponges”. They can influence various biological processes, including cell differentiation, cell proliferation, apoptosis, and stress response [41,42]. Additionally, due to their circular structure, circRNAs are more resistant to RNAse degradation than linear ncRNAs [43].

CircRNAs can contain complementary binding sequences for specific proteins, allowing them to directly interact with these proteins, forming stable complexes and preventing them from carrying out their normal cellular functions [44].

It has been reported that circRNAs are abundantly expressed in brain tissue, where they play an important role in neural development [45].

Given this statement, it could be interesting to investigate the role of ncRNAs as diagnostic and prognostic indicators, thus contributing to our understanding of their role in brain damage. In this review, we summarize the studies that have shown the involvement both of lncRNAs and circRNAs in brain damage related to hypoxic/anoxic conditions.

## 2. Methodology

**Figure 1:**
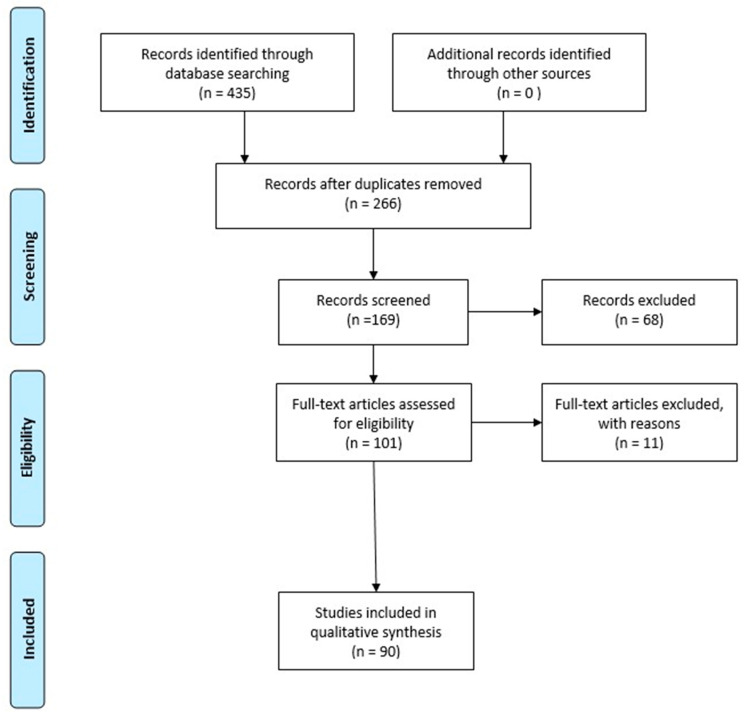
PRISMA flow diagram illustrating the selection methodology of the studies used to draft the review. Duplicate articles were excluded from the total number of studies recorded. Instead, articles evaluating the involvement of long noncoding RNAs and circular RNAs in hypoxic/anoxic brain damage were selected. The PRISMA Statement is published in [46].

Publications between 2017 and 2023 were taken into consideration for this review. PubMed was used to retrieve the publications corresponding to the keywords: “Long non-coding RNA” and “hypoxic brain damage” (135 articles), “circRNAs” and “hypoxic brain damage” (32 articles), Long non-coding RNA” and “anoxic brain damage” (128 articles), “circRNAs” and “anoxic brain damage” (28 articles), “Long non-coding RNA” and “ischaemic hypoxic brain injury” (92 articles), and “circRNAs” and “ischaemic hypoxic brain injury” (20 articles). Studies regarding the roles of long non-coding RNAs and circRNAs in hypoxic/anoxic brain damage were selected. We considered 90 studies (Figure 1).

## 3. LncRNAs and circRNAs in Hypoxic Brain Damage

LncRNAs differentially expressed both spatially and temporally and specific to certain brain regions, alongside transcription factors and epigenetic regulators, constitute important components of CNS regulatory gene networks. LncRNAs are recognized as key regulators in differentiation, neuronal function, and brain development. Dysfunction or dysregulations in lncRNAs could potentially be implicated in neurological disorders such as hypoxic-ischemic brain damage and cerebral ischemia reperfusion injury [36,47]. Given that the brain is the major consumer of oxygen, it is closely linked to the risk of hypoxia-induced neurological insults, which, in turn, are associated with many CNS diseases. Hence, therapeutic strategies based on protective lncRNAs hold promise in mitigating brain damage induced by ischemic/hypoxic conditions [48]. Under hypoxic conditions, cells endure prolonged energy deprivation leading to irreversible cellular dysfunction and, ultimately, cellular death [49]. Consequently, hypoxic conditions trigger several reactions, including deficits in ion channel homeostasis, free radical production, and energy failure. In this regard, following hypoxia, a reduction in intracellular ATP/ADP ratios has been demonstrated, leading to a loss of concentration gradients induced by the increased efflux of K^+^ ions and influx of Na^+^ and Ca^2+^ ions, as well as consequent membrane depolarization [50]. Therefore, membrane depolarization and increased Ca^2+^ ions lead to protease activation, resulting in membrane damage, deficits in mitochondrial metabolism, and the generation of reactive oxygen species (ROS) [51]. Prolonged exposure to hypoxia can upregulate hypoxia-inducible factor (HIF), widely regarded as a potential endogenous marker of hypoxia [52]. Hence, it has become evident that vulnerability to hypoxia-induced diseases increase with age. Indeed, it has been shown that, with age, oxygen pressure decreases, which, combined with lower cerebral perfusion, can lead to neuroinflammation and microglial activation [53,54]. It is noteworthy that age-related changes in HIF have been associated with an increased susceptibility for stroke [55].

Therefore, therapeutic strategies involving lncRNAs could be potentially useful for improving brain damage induced by ischemic/hypoxic conditions in the CNS.

CircRNAs play important roles during mammalian brain development [56]. They are highly abundant in several regions of the brain such as the cerebellum, cortex, striatum, olfactory bulbs, and hippocampus [38,57,58]. Studies have demonstrated that circRNAs can alter the function of miRNAs after a stroke, leading to changes in the regulation of their target mRNAs [59]. These miRNAs, in turn, could play an significant role in hypoxic/anoxic conditions.

## 4. Studies about lncRNAs in Hypoxic/Anoxic Conditions

### 4.1. SNHG Family in Hypoxic/Anoxic Conditions

The small nucleolar RNA host gene (SNHG) family comprises genes that contain and encode small nucleolar RNA (snoRNA) within their genomic regions. SnoRNAs play a crucial role in gene expression regulation and RNA maturation [60].

The SNHG family has been associated with osteoblast differentiation [61] and found to be dysregulated in various human diseases, including neonatal pneumonia, diabetic retinopathy, neuropathic pain, acute cerebral infarction, acute myeloid leukemia, endometriosis, glioblastoma, neuroblastoma, and cervical cancer [62].

Studies have shown that lncRNA SNHG1 reduces the effect of oxygen-glucose deprivation (OGD) in cells, such as brain microvascular endothelial cells (BMECs) and SH-SY5Y, by regulating miR-140-5p [63] and miR-338 [64], thereby modulating the HIF-1α/ vascular endothelial growth factor (VEGF)-A pathway. The results demonstrate that this lncRNA plays a crucial role in angiogenesis and neuronal protection, as indicated by the levels of caspase 3, Bax, B-cell lymphoma 2 (Bcl-2), and Bcl-XL.

Instead, Yang et al. delved into the role of Snhg3 in hypoxic ischemic brain damage (HIBD) pathogenesis. They observed the downregulation of this lncRNA in both primary hippocampal cells from mice and neonatal C57Bl/6 mice subjected to hypoxic conditions, mimicking HIBD. In vitro experiments unveiled that Snhg3 acts as a sponge for miR-196, thereby influencing the expression of target genes, including XIAP and CAAP1, which are involved in apoptosis and, consequently, hippocampal cell function. The overexpression of Snhg3 was shown to decrease apoptosis in hippocampal cells, as evidenced by increased cell viability. Consistent with these findings, in vivo experiments revealed that Snhg3 overexpression improved neurological function in animals and reduced HIBD, as indicated by the reduced astrocytosis, microgliosis, and brain infarct size [65].

Cheng et al. evaluated the role of small nucleolar RNA host gene 12 (SNHG12) in oxygen-glucose deprivation/reperfusion (OGD/R)-induced primary neurons. Additionally, it was reported that SNHG12 knockdown exacerbated apoptosis induced by OGD/R conditions, as shown by the Bcl-2/Bax ratio, and also increased pro-inflammatory factors, including Monocyte chemoattractant protein-1, E-SELECTIN, and interleukin-6 (IL-6). Conversely, it was highlighted that SNHG12 led to a reduction in OGD/R injury through Akt pathway modulation [66] and increased cell viability via the modulation of SIRT1/FOXO3a signaling, as also demonstrated by Wu et al. using an I/R model of HT22 cells [67].

Through the use of OGD and middle cerebral artery occlusion/reperfusion (MCAO/R) models, it has been observed that SNHG14 is overexpressed under hypoxic conditions. This lncRNA reduces the levels of miR-98-5p [68] and miR-181c-5p [69] by modulating BCL2L13 and BMF. SNHG14 silencing reduces apoptosis and inflammation and promotes cell proliferation, exerting neuroprotective effects.

The research group of Fu et al. investigated the effects and mechanisms of lncRNA Small Nucleolar RNA Host Gene 15 (SNHG15) on HI brain injury. They used a mouse model of hypoxic/ischemic (H/I) brain injury and an OGD model established in PC12 cells. This study revealed that SNHG15 was increased in both models. Furthermore, they analyzed the SNHG15/miR-153-3p/SETD7 axis and demonstrated that the suppression of SNHG15 alleviated HI brain injuries by modulating this axis. Therefore, for HI, this lncRNA could be a potential therapeutic target [70].

**Figure 2:**
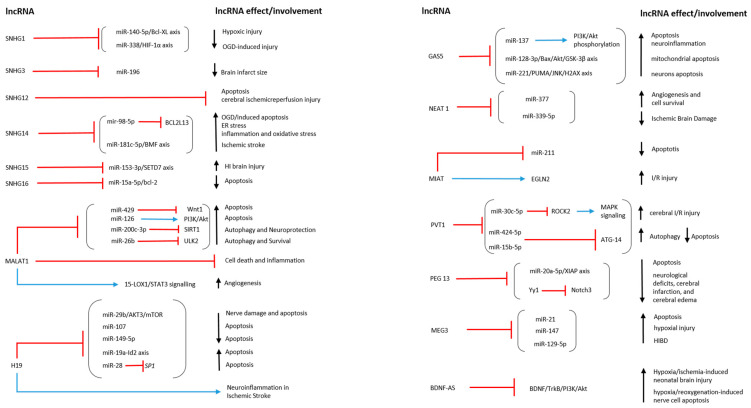
This image illustrates the action mechanism and involvement of lncRNAs in hypoxic/anoxic conditions. The red line represents the inhibition symbol, while the blue line represents the activation symbol. ↑: increase; ↓: decrease [63,64,65,66,67,68,69,70,71,72,73,74,75,76,77,78,79,80,81,82,83,84,85,86,87,88,89,90,91,92,93,94,95,96,97,98,99,100].

**Figure 3:**
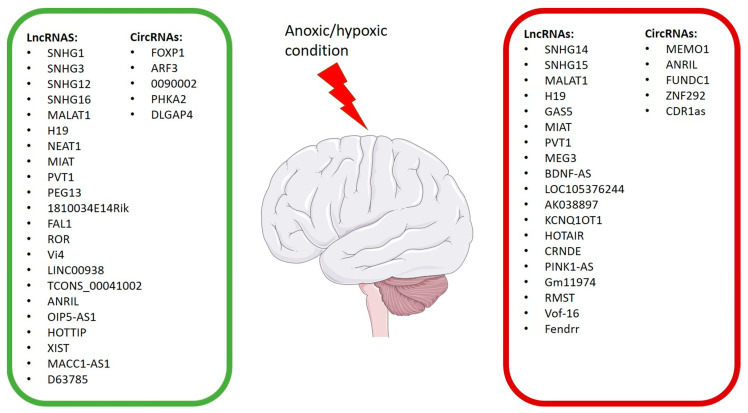
LncRNAs and CircRNAs affected from anoxic/hypoxic conditions. The green rectangle contains the LncRNAs and CircRNAs that improve hypoxic/anoxic conditions; the red rectangle contains the LncRNAs and CircRNAs that worsen hypoxic/anoxic conditions.

Teng et al. investigated the role of lncRNA SNHG16 in an OGD/R model using human brain microvascular endothelial cells (HBMECs). To evaluate cell viability, they used flow cytometry as the main indicator of apoptosis. They also demonstrated that lncRNA SNHG16, by directly interacting with miR-15a-5p, inhibited apoptosis and promoted proliferation. The overexpression of SNHG16 led to a decrease in miR-15a-5p and an increment in Bcl-2 expression levels, as well as reducing apoptosis and enhancing cell survival [71] (Table 1; Figure 2 and Figure 3).

Based on the provided information, several SNHG family members have been studied in the context of ischemic and hypoxic brain injuries, having a significant impact on cell survival, angiogenesis, apoptosis, and inflammation in the brain. For instance, SNHG1 has been implicated in regulating the HIF-1α/VEGF-A pathway and neuronal protection, while SNHG3 acts as a sponge for miR-196, influencing apoptosis-related genes.

Additionally, other members like SNHG12 and SNHG14 have demonstrated the modulation of apoptosis and inflammation through specific signaling pathways such as the Akt pathway and the regulation of miR-98-5p and miR-181c-5p.

Furthermore, SNHG15 and SNHG16 have been linked to cell survival and neuronal protection by interacting with specific microRNAs and target genes such as SETD7 and Bcl-2.

In summary, studies on the SNHG family have clearly indicated their role in neuroprotection, suggesting that their regulation could be a promising therapeutic strategy for ischemic and hypoxic brain injuries.

### 4.2. LncRNA MALAT1 in Hypoxic/Anoxic Conditions

Metastasis-Associated Lung Adenocarcinoma Transcript 1 (MALAT1) is an lncRNA initially associated with lung cancer [101]. However, it has recently gained interest in neurological research due to its involvement in nuclear organization and the regulation of apoptosis. MALAT1 is linked to neurological disorders such as Alzheimer’s [102], Parkinson’s [103], and autism [104]. Studies have also associated MALAT1 with hypoxic/anoxic conditions; it increases under H/I conditions, as observed in OGD models, and acts as a sponge for several miRNAs, including miR-429, miR-200c-3p, and miR-126. These interactions have various effects on cell survival. The sequestration of miR-429 [72] and miR-126 [73] leads to an increase in WNT1 expression and suppresses the PI3K/Akt pathway, worsening the hypoxic condition. However, through its interaction with miR-200c-3p and the subsequent increase in SIRT1 expression levels, MALAT1 promotes autophagy and could protect against OGD-induced conditions [74].

The autophagy activity was confirmed in the study by Li et al. in OGD/R-exposed C57BL/6J mice BMECs, demonstrating that MALAT1 was upregulated after OGD/R conditions, leading to an increase in autophagy and cell survival in BMECs. Moreover, they evaluated the mechanism through which MALAT1 exerted these effects. Indeed, it was discovered that MALAT1, by directly binding to miR-26b, reduced its expression. On the other hand, the overexpression of miR-26b, through its targeting of ULK2, inhibited the BMECs’ autophagy and survival under OGD/R-induced conditions. The study results demonstrated that MALAT1 acts as a ceRNA, modulating miR-26b and upregulating ULK2 expression [75].

MALAT1 may protect the brain’s microvascular and parenchymal components from ischemic damage via anti-apoptotic and anti-inflammatory molecular mechanisms. Zhang et al. observed that MALAT1 was upregulated in both mouse BMECs and the cerebral microvessels of MALAT1 knockout (KO) mice following exposure to OGD or intraluminal MCAO, respectively. In particular, the authors showed that MALAT1 interacted with Bim and E-selectin, effectively reducing cell death and inflammation. Moreover, ischemic brain injury was aggravated after silencing MALAT1 with fluorescein-labeled MALAT1 GapmeR, both in in vitro and in vivo conditions [76].

Also, Wang et al., using the similar in vitro and in vivo models, investigated the role of MALAT1. Both the in vivo and in vitro results reported an increase in angiogenesis and a significant overexpression of MALAT1 following ischemic injury. Notably, the same trend was observed between the expression of MALAT1 and the angiogenesis-associated marker CD31 in EC proliferation. MALAT1 was found to promote cerebral EC proliferation and migration, as indicated by CD31-positive cells after OGD/R. Additionally, the authors discovered that MALAT1 regulated angiogenesis through the 15-lipoxygenase 1 (LOX1)/Signal transducers and activators of transcription 3 (STAT3) signaling pathway activation, confirming its role in the proliferation and migration of brain microvascular cells after ischemic injury [77] (Table 1; Figure 2 and Figure 3).

In summary, MALAT1’s regulatory roles in autophagy, apoptosis, inflammation, and angiogenesis position it as a potential therapeutic target for mitigating cellular damage in hypoxic/ischemic brain conditions.

### 4.3. LncRNA H19 in Hypoxic/Anoxic Conditions

LncRNA H19 plays a significant role in regulating various biological processes. Initially identified as a highly expressed lncRNA during embryonic development, H19 is associated with the insulin-like growth factor 2 (IGF2) gene [105].

There are conflicting findings regarding H19, as various studies have associated the overexpression of this lncRNA with improved hypoxic conditions. For instance, in the study by Zhu et al. on neonatal Sprague Dawley (SD) rats with hypoxic-ischemic encephalopathy (HIE), they demonstrated the neuroprotective mechanism of H19 in reducing autophagy through the miR-29b/Akt3/mTOR pathway [78]. It was also observed that its overexpression is correlated with increased VEGF expression and, simultaneously, reduced miR-107 expression, leading to a reduction in apoptosis and cognitive deficits [79].

Additionally, Zhang et al. investigated the changes in H19 expression levels in a neonatal rat model of HIBD. They observed a decrease in H19 expression. However, H19 overexpression reduced inflammation and oxidative stress in the in vivo experimental model. Furthermore, the involvement of miR-149-5p, activating the PI3K/Akt signaling pathway, was noted [80].

Conversely, a significant study by Xiao et al. identified the H19-miR-19a-Id2 axis as a potential novel target for ischemic brain injury therapy. The H19 levels increased in blood samples of ischemic stroke (IS) patients, the penumbra area of MCAO/R rats, and OGD-treated neuronal cells. The inhibition of H19 reduced neuronal apoptosis induced by hypoxia/ischemia both in vivo and in vitro [81].

Chen et al. observed that H19 overexpression increased cell damage induced by hypoxic conditions, while H19 silencing alleviated neuronal damage. The authors demonstrated that H19 targets miR-28, regulating its expression. MiR-28, in turn, targets the transcription factor SP1, involved in angiogenesis, growth, and cell survival, thereby promoting the activation/deactivation of the PDK/AKT and JAK/STAT signaling pathways [82].

The effects of lncRNA H19 were demonstrated by Wang et al. in IS patients and an MCAO mouse model of transient focal cerebral ischemia. The study revealed upregulated circulating H19 levels in the IS patients. Additionally, in the MCAO mice model, the H19 levels were positively related to the severity of neurological deficits, also leading to increased tumor necrosis factor (TNF)-α and IL-1β levels in brain tissue and plasma. Conversely, H19 silencing reduced infarct volume, brain edema, and inflammation. It also improved neurological deficits 24 h after the in vivo model induction. To explain the role of H19 in microglia reprogramming after ischemia, Wang et al., through the use of BV2 cell OGD exposure, demonstrated that H19 silencing led to microglia changes from M1 to M2 polarization according to a mechanism that involved histone deacetylase 1 (HDAC1) regulation. Therefore, the study reported that H19 could be a useful marker as well as a potential therapeutic target for IS patients [83] (Table 1; Figure 2 and Figure 3).

In summary, the role of the lncRNA H19 in hypoxic/ischemic conditions is complex. While some studies have proposed a protective effect through the regulation of various signaling pathways and interactions with different microRNAs, others have indicated a detrimental role in cellular damage and brain inflammation. Further research is needed to fully understand its function and therapeutic potential in brain hypoxic/ischemic pathologies.

### 4.4. LncRNA GAS5 in Hypoxic/Anoxic Conditions

As neuroinflammation and apoptosis are commonly known hallmarks involved in H/I events, it could be useful to investigate how the modulation of lncRNAs improves them. Jing et al. linked the lncRNA Growth Arrest-Specific 5 (GAS5)/miR-137 axis to cardiopulmonary cerebral resuscitation (CCR). They showed that GAS5 silencing or miR-137 overexpression activate the PI3K/Akt pathway, leading to the inhibition of apoptosis and neuroinflammation during acute systemic hypoxia/reoxygenation (H/R) injury [84].

Additionally, Wang et al. discovered a relationship between lncRNA GAS5 and the miR-128-3p/Bax/Akt/GSK-3β axis. In detail, they showed that the inhibition of lncRNA GAS5 suppressed mitochondrial apoptosis in neonatal HIBD rats [85].

The role of GAS5, as well as its involvement in cerebral ischemia, was also evaluated by Zhou et al. They observed that GAS5 was upregulated in MCAO SD rats and hypoxia-induced cell models, including primary rat cortical cells and B35 cells, leading to an increment in apoptosis. Also, they reported that miR-221 was downregulated in in vitro models. It was noteworthy that GAS5, through miR-221 sponging, led to the modulation of the p53-upregulated modulator of apoptosis (PUMA), which is involved in apoptosis, and JNK/H2AX signaling. Overall, the study revealed that, in hypoxic conditions, GAS5 worsens apoptosis by targeting the miR-221/PUMA axis [86] (Table 1; Figure 2 and Figure 3).

In summary, these studies emphasized the intricate involvement of lncRNAs, particularly GAS5, in regulating apoptosis and neuroinflammation during H/I events. The findings suggest potential therapeutic targets, highlighting the need for further investigation to develop effective interventions for hypoxic/ischemic brain injuries.

### 4.5. LncRNA NEAT1 in Hypoxic/Anoxic Conditions

Nuclear paraspeckle assembly transcript 1 (NEAT1) is an lncRNA transcribed from the familial tumor syndrome multiple endocrine neoplasia (MEN). It has been associated with tumor progression, acting as a cancer driver factor [106].

The role of this lncRNA in apoptosis, as well as its possible involvement in angiogenesis, was studied by Zhou et al. in mouse primary BMECs that were OGD-induced. The authors reported that NEAT1, by targeting miR-377, led to the overexpression of VEGFA, BCL-XL, and SIRT1, improving both the angiogenesis and viability of BMECs. Conversely, NEAT1 silencing resulted in the inhibition of angiogenesis and exacerbated apoptosis in the OGD-induced BMECs. Therefore, NEAT1 silencing upregulated miR-377 and downregulated its downstream targets, including VEGFA, SIRT1, and BCL-XL, thus inhibiting angiogenesis and cell survival [87].

Additionally, Zhao et al. evaluated the role of NEAT1 using a neonatal HIBD mouse model induced by ligation of the right common carotid artery and an OGD-induced neuronal cell model. The researchers investigated the lncRNA NEAT1 by overexpressing and knocking it down, aiming to assess its impact on miR-339-5p, which was overexpressed in both HIBD experimental models. The study findings demonstrated that NEAT1 alleviates HIBD in mice by binding to miR-339-5p [88] (Table 1; Figure 2 and Figure 3).

These findings underscore NEAT1’s significant role in regulating angiogenesis, cell survival, and apoptosis under H/I conditions, offering potential therapeutic targets for hypoxic/ischemic brain injuries

### 4.6. LncRNA MIAT in Hypoxic/Anoxic Conditions

Myocardial Infarction Associated Transcript (MIAT) lncRNA was initially discovered in Japanese patients who suffered from myocardial infarction in a genome-wide association study [107].

Li et al. investigated the role of this lncRNA in HIBD using OGD-induced Neuro2A cells and neonatal rats with permanent unilateral carotid ligation as experimental models. They discovered that the overexpression of MIAT reduced neuron apoptosis and alleviated HI injury in the neonatal rats through the miR-211/GDNF pathway, demonstrated by a reduction in caspase-3 levels [89].

Another research group led by Li et al. demonstrated that lncRNA MIAT modulated cerebral I/R injury in MCAO SD rats and primary rat neurons that were OGD/R-exposed. This modulation was achieved through the MIAT-EGLN2 axis. The study results showed that MIAT exacerbated I/R injury by disrupting the redox homeostasis in neurons. MIAT overexpression led to an increased stability of EGLN2, reducing its ubiquitin mediation through the degradation of MDM2-mediated K48 poly-ubiquitination [90] (Table 1; Figure 2 and Figure 3).

In summary, MIAT exhibits a dual role in hypoxic/ischemic brain injuries: it provides neuroprotection by reducing apoptosis via the miR-211/GDNF pathway in certain contexts, while exacerbating injury through the disruption of redox homeostasis in others. Understanding these mechanisms could pave the way for targeted interventions in hypoxic/ischemic brain disorders.

### 4.7. LncRNA PVT1 in Hypoxic/Anoxic Conditions

LncRNA PVT1 (plasmacytoma variant translocation 1) was discovered through studies conducted on renal cell carcinoma cells, where it was found to be overexpressed and highly dysregulated compared to other solid tumors [108,109].

Zhang et al. demonstrated that silencing PVT1 reduced infarct volume and improved neurological behavior in MCAO mice. In the same way, a reduction in apoptosis and an increase in cell viability were reported in primary brain neurons induced by OGD/R. Noteworthy, PVT1 acts as a ceRNA for miR-30c-5p, modulating Rock2, which activates the mitogen-activated protein kinase (MAPK) pathway and exacerbates cerebral I/R injury [91].

Additionally, Zhang et al. demonstrated that PVT1 alleviated hypoxia-induced endothelial apoptosis via the miR-15b-5p/ATG14 and miR-424-5p/ATG14 axis, increasing the autophagy levels in human umbilical vein endothelial cells (HUVECs) that were OGD-induced. Instead, silencing PVT1 suppressed cellular autophagy, promoting apoptosis under OGD conditions. PVT1 also induced autophagy in HUVECs by regulating ATG14 miRNA, as demonstrated by RT-qPCR results [92] (Table 1; Figure 2 and Figure 3).

In conclusion, lncRNA PVT1 plays a crucial role in regulating apoptosis and autophagy during hypoxic/ischemic brain injuries. Its interaction with different signaling pathways underscores the significance of PVT1 as a potential therapeutic target for hypoxia/ischemia-related neurological conditions.

### 4.8. LncRNA PEG13 in Hypoxic/Anoxic Conditions

LcnRNA Peg13 has been characterized in relation to important imprinting mechanisms associated with potassium ion channels in the brain [110].

The neuroprotective effects of this lncRNA in the progression of HIBD were explored by Gao et al. in neonatal HIBD mice and mouse hippocampal neurons exposed to OGD. The authors revealed that lncRNA Peg13 exerts anti-apoptotic effects by acting as a sponge for miR-20a-5p, which targets the X chromosome-linked inhibitor of apoptosis (XIAP) in an in vitro model. In addition, it was demonstrated that, Peg13, through the miR-20a-5p/XIAP axis, contributed to a reduction in HIBD severity in neonatal mice [93].

Li et al. also found that lncRNA Peg13 and Gli2 expression decreased in bEnd.3 cells treated with OGD/R. They showed that this lncRNA has neuroprotective effects through the Gli2/Peg13/Ezh2/Yy1/Notch3 axis. It is noteworthy that the overexpression of Notch3 inhibited OGD/R-induced endothelial dysfunction. Using an I/R injury model, they also demonstrated that Peg13 or Gli2 overexpression reduces I/R-induced neurological deficits, cerebral infarction, and cerebral edema [94] (Table 1; Figure 2 and Figure 3).

In summary, Peg13 exhibits anti-apoptotic and neuroprotective properties in the context of hypoxic-ischemic brain damage. Its interaction with the miR-20a-5p/XIAP axis and involvement in the Gli2/Peg13/Ezh2/Yy1/Notch3 pathway highlights its potential as a therapeutic target for HIBD-related conditions.

### 4.9. LncRNA MEG3 in Hypoxic/Anoxic Conditions

Maternally expressed gene 3 (MEG3) lncRNA, which is approximately 1.7 kb, is expressed at high levels in brain tissues and it has also been related to the suppression of the proliferation of cancer cell lines [111,112].

Deng et al. investigated the involvement of this lncRNA in HIBD. In detail, they evaluated the role of lncRNA MEG3 in rat PC12 cells exposed to 24 h of hypoxia. The in vitro results demonstrated that, MEG3 silencing, performed using siRNA, exerted a neuroprotective function, preventing injury-induced hypoxia. This was achieved through the modulation of several proteins involved in apoptosis and cell proliferation, including Bax, caspase-3, CyclinD1, p53, and p21. Interestingly, MEG3 acts as a miR-21 sponge, leading to the activation of the PI3K/AKT pathway and the inhibition of nuclear factor kappa B (NF-κB) pathway. Moreover, when miR-21 and MEG3 were simultaneously silenced, the neuroprotective effect was reduced, emphasizing the significance of the MEG3-miR-21 axis. Interestingly, the research group led by Yan et al. approached the study of MEG3 in different conditions. They found a direct connection between MEG3 and miR-21, overexpressing the latter. Indeed, the results showed that miR-21 protect cells from OGD/R induced apoptosis. In order to better understand how MEG3 exerted its effects, they also found that miR-21 competes with programmed cell death 4 (PDCD4) mRNA, creating a ceRNA which is able to regulate ischemic neuronal death. To further emphasize the biological functions of MEG3, they also silenced the lcnRNA in a focal cerebral ischemia and reperfusion model induced by intraluminal MCAO. Indeed, they found that silencing MEG3 leads to protection against in vivo I/R-induced ischemic damage and improved overall neurological functions [95,96].

In another study, Hen et al. found that MEG3 was upregulated in PC12 cells under hypoxic conditions. The authors demonstrated that the overexpression of MEG3 led to increased cell damage during hypoxia, while transfecting cells with short-hairpin RNA against MEG3 alleviated the injury. The study also revealed that MEG3 inhibited miR-147 by binding its 3′-UTR, suppressing Sox2 expression. Notably, Sox2 was associated with cell viability via the NF-Kb and Wnt/β-catenin pathways. Consequently, MEG3 increased the hypoxic damage in PC12 cells via the targeting of miR147 and the downstream Sox2 [97].

The interaction between the lncRNA MEG3 and the therapeutic effect of dexmedetomidine (DEX) on HIBD was studied by Zhou et al. They found that MEG3 targets and inhibits miR-129-5p. Furthermore, it was demonstrated that silencing MEG3 enhances the therapeutic effect of DEX on HIBD in neonatal mice by increasing the expression of miR-129-5p [98] (Table 1; Figure 2 and Figure 3).

In summary, MEG3 appears to play a pivotal role in the regulation of apoptosis and cell survival during HIBD. Its interaction with miR-21, miR-147, and miR-129-5p highlights its potential as a target for therapeutic intervention in hypoxic-ischemic brain damage.

### 4.10. LncRNA BDNF-AS in Hypoxic/Anoxic Conditions

HIBD stands as one of the major causes of acute neonatal brain injury that leads to high mortality rate and severe neurological deficits. Brain-derived neurotrophic factor (BDNF) is known to be involved in various neurological processes, attracting a lot of interest from researchers. Qiao et al. investigated the role of lncRNA BDNF Antisense RNA (BDNF-AS) in neonatal HIBD in order to comprehend the underlying mechanism mediated by BDNF-AS in brain injury. The in vitro results demonstrated that BDNF-AS silencing led to a reduction in cell apoptosis. Similarly, the study was conducted on male pups of C57BL/6J mice, where, in line with the in vitro results, BDNF-AS silencing resulted in an increase in BDNF expression [99].

The role of lncRNA BDNF-AS in neuronal apoptosis induced by H/R was also evaluated by Zhong et al. both in human cortical neurons (HCN2) and human astrocytes. It was found that, following H/R, the expressions of BDNF, p-AKT/AKT, and TrKB decreased, while that of BDNF-AS was upregulated. The authors reported that BDNF-AS knockdown, performed using siRNA, suppressed apoptosis, reduced mitochondrial membrane potential, and led to increased BDNF in both cell models that were H/R-induced. Overall, the study results demonstrated that BDNF-AS siRNA exerted a neuroprotective effect through the activation of the BDNF–TrkB–PI3K/Akt pathway. Therefore, lncRNA BDNF-AS could be an important target in brain damage induced by H/R [100] (Table 1; Figure 2 and Figure 3).

Based on the findings from the studies conducted by Qiao et al. and Zhong et al., it appears that BDNF-AS plays a significant role in neonatal brain cell apoptosis induced by hypoxic-ischemic brain injury (HIBD). These results suggest that BDNF-AS silencing has a neuroprotective effect through the activation of the BDNF–TrkB–PI3K/Akt pathway. Consequently, BDNF-AS could be a promising target for reducing brain damage caused by H/R. In summary, these results indicate that BDNF-AS might be a promising area of research for the development of targeted therapies against neuronal apoptosis associated with neonatal brain damage caused by hypoxic-ischemic injury.

### 4.11. Other lncRNAs That Play a Role in Exacerbating Hypoxic/Anoxic Conditions

Using the OGD/R model and MCAO in mice, researchers analyzed the effect of lncRNAs after this type of specific injury. Several lncRNAs have been identified to play a role in injury caused by hypoxic/anoxic conditions through miRNA sponging.

For instance, miR-145 expression was significantly increased in the blood of IS patients, and EPHA4 was identified as a target of miR-145. Furthermore, Cai. et al. demonstrated that the lncRNA LOC105376244 was regulated by miR-145–EPHA4 interaction [113].

In contrast, Wei et al. observed an increase in the lncRNA AK038897 levels in hypoxic conditions and showed that this lncRNA binds to miR-26a-5p, regulating its expression. This miRNA, in turn, directly targets DAPK1, suggesting that AK038897 plays a role in cerebral I/R injury through the AK038897/miR-26a-5p/DAPK1 signaling cascade, which acts as a regulator of ischemic neuronal death [114]. Several lncRNAs exacerbate or contribute to hypoxic conditions, including Potassium voltage gated channel subfamily Q member 1 opposite strand 1 (KCNQ1OT1) lncRNA. The results of Yi et al. study suggested that KCNQ1OT1 aggravates cerebral ischemia/reperfusion injury (CI/RI) by binding to miR-140-3p, disrupting its interaction with HIF-1α [115].

Another important enhancer of hypoxia induction was investigated by Wang et al., where the increased expression of the lncRNA HOTAIR was observed in the plasma of neonatal HIE patients and in an in vitro model of OGD/R injury using hBMVECs. HOTAIR was found to interact with EZH2, and the study suggested that the HOTAIR/EZH2 axis may exert an important role in causing damage to the tight and adherens junctions structure through the downregulation of ZO-1, occludin, clau-din 5, and VE-cadherin expression. Consequently, HOTAIR is suspected to play a role in the injury and apoptosis of hBMVECs, likely leading to blood–brain barrier (BBB) damage, mediated by EZH2 [116].

Fu et al. investigated the role of colorectal neoplasia differentially expressed (CRNDE), an intergenic lncRNA located on chromosome 16, involved in neurogenesis and normal brain development. They study demonstrated that an intracerebroventricular injection of lentiviral vector expressing CRNDE shRNA led to a reduction in infarct volume and apoptosis, as well as improving memory and learning deficits in the SD rats brain exposed to HIBD, thus confirming the negative role that CRNDE holds in this pathological context [117].

Yang et al. demonstrated that PINK1-AS accelerates oxidative stress induced by CI/RI by functioning as a sponge for miR-203.The PINK1-AS/miR-203/ATF2 axis was found to be involved in the regulation of NOX2 expression, contributing to the cellular response to oxidative stress [118].

In their study, Cai et al. knocked down the lncRNA Gm11974, demonstrating its neuroprotective effect by alleviating apoptosis in OGD-exposed N2a cells. The results suggest that this protective effect is mediated by the miR-766-3p/NR3C2 axis [119].

The study conducted by Zhao et al. aimed to analyze the functional mechanism and role of the lncRNA RMST. Using qRT-PCR, the authors focused on the expression levels of RMST, miR-377, and Semaphorin 3A (SEMA3A), along with their respective changes. Their findings indicated that silencing RMST prevented OGD/R-triggered apoptosis and oxidative stress. RMST blockage resulted in an increase in miR-377 levels because RMST acts as a sponge for miR-377. Consequently, this miRNA reduction led to decreased SEMA3A levels. Moreover, the downregulation of SEMA3A enhanced cell viability and reduced cell apoptosis [120].

On the other hand, Tan et al. investigated the role of the lncRNA Vof-16 in HIE neonatal SD male rats. Knocking out Vof-16 promoted the recovery of neuronal damage and neurobehavioral function [121].

In another study, Wang et al. examined lncRNA-Fendrr and suggested its potential utility against diabetic cerebral I/R injury. This lncRNA played a crucial role in inflammation. Specifically, a reduction in lncRNA-Fendrr led to decreased levels of NLRC4, ASC, pro-caspase-1, and caspase-1-mediated pyroptosis in a diabetes-cerebral I/R BV-2 cell model. Fendrr resulted in being able to protect against the degradation of NLRC4 protein through the E3 ubiquitin ligase HERC2, thereby increasing pyroptosis in the microglia [122] (Table 2 and Figure 2).

In summary, these studies indicate that numerous lncRNAs play critical roles in ischemic brain injuries by engaging in complex interactions with miRNAs and signaling pathways, which strongly need to be further understood in order to deeply exploit them as potential therapeutic targets for conditions associated with ischemic brain injuries. Further research could lead to targeted therapeutic developments based on these lncRNAs to protect the brain from ischemic damage.

### 4.12. Other lncRNAs Possessing Neuroprotective Properties

In the studies we examined, several lncRNAs showed neuroprotective effects against hypoxic/anoxic conditions through the modulation of a plethora of signaling pathways.

For instance, Zhang et al. explored the role of lncRNA-1810034E14Rik both in vivo and in vitro. Their results revealed that its overexpression inhibited of the NF-κB pathway, leading to a reduction in inflammatory cytokine levels, mitigating brain damage [123].

Gao et al. investigated the impact of lncRNA FAL1. They found that its overexpression reduced inflammation, as evidenced by the decreased levels of IL-6 and monocyte chemotactic protein-1 (MCP-1). Furthermore, FAL1 restoration resulted in elevated levels of phosphorylated PAK1/AKT and proliferating cell nuclear antigen (PCNA), which had been diminished by OGD/R exposure. FAL1 also modulated the nitric oxide synthase (eNOS)/nitric oxide (NO) and PAK1/AKT signaling pathways, highlighting its multifaceted role beyond its known association with cancer development [124].

Zhou et al. discovered a connection between the lncRNA regulator of reprogramming (ROR) and hypoxia. The in vivo findings demonstrated a significant increase in ROR levels and, in turn, inhibited TNF-α/P-ASK-1-mediated apoptosis in the in vitro model. This effect was achieved through an enhanced ASK-1/STRAP/14-3-3 complex, ultimately alleviating hypoxia-induced damage in brain tissue [125].

Xiong et al. focused on a novel lncRNA called TCONS00044054 (Vi4) and its interactions. Using SD neonatal rats and a subset of neonatal miR-185-5p KO rats, they studied an HIE model to understand the role of this lcnRNA. Their research revealed a crucial interaction between Vi4 and miR-185-5p. Silencing miR-185-5p improved long-term motor functions and reduced deficits in learning and memory in HIE rats. Conversely, Vi4 overexpression enhanced these conditions by boosting the transcription of Insulin-like growth factor-binding protein 3 (Igfbp3) and its downstream functions. Vi4 acted as a positive regulator of neuronal survival, encouraging a reduction in cell apoptosis [126].

Zhao et al. demonstrated that LINC00938 acts as a neuroprotective factor against neonatal brain injury induced by HIE. Their study, involving 16 infants (8 with HIE and 8 controls), showed reduced RNA levels of LINC00938 in HIE patients and in an in vitro model. This specific lncRNA promoted neuronal cell survival under hypoxic conditions by inhibiting the activation of the p-JNK and p-p38 MAPK signaling pathways [127].

Additionally, Xiong et al. highlighted the neuroprotective role of TCONS_00041002 overexpression. It led to a reduction in brain infarction size and promoted neuron survival, resulting in an overall improvement in rats’ neurological conditions [128].

Similarly, there are lncRNAs that exert a role in hypoxic/anoxic conditions. Some of these lncRNAs have a neuroprotective role achieved through miRNA sponging, as demonstrated in the study by Li et al., where they investigated the role of the lncRNA ANRIL. They found an increased ANRIL expression in both cell types during hypoxia. Conversely, the siRNAs suppression of ANRIL reduced cell viability and increased apoptosis. ANRIL was also found to act as a sponge for miR-378b, regulating the expression of autophagy-related 3 (ATG3). Thus, ANRIL has a neuroprotective effect against apoptosis by enhancing autophagy and reducing neuronal cell death [129].

As well as in the previous paper, Chen et al. demonstrated that lncRNA OIP5-AS1 exerts neuroprotective effects by acting as a sponge for miR-186-5p, thereby activating CTRP3 [130].

In the wake of these considerations, Wang et al. evaluated the expression of the HOXA transcript at the distal tip (HOTTIP) and miR-143 both in vivo and in vitro. They observed a reduction in HOTTIP and an increase in miR-143 expression in both models. Furthermore, they demonstrated that HOTTIP may function as a competing endogenous RNA (ceRNA) by sponging miR-143. Interestingly, the overexpression of HOTTIP increased cell viability, reduced apoptosis, and upregulated the expression of hexokinase 2 (HK-2), pyruvate kinase M2, and glucose uptake. HOTTIP overexpression also improved glycolytic metabolism by upregulating HK-2 expression and reduced neuronal injury [131].

Considering the importance of angiogenesis in recovery from ischemic stroke (IS), Hu et al. investigated the role of the lncRNA X-inactive specific transcript (XIST) in human brain microvascular endothelial cells (HBMECs) under hypoxic conditions. Their study revealed the upregulation of XIST in HBMECs, enhancing cell proliferation, migration, and angiogenesis. Additionally, XIST competitively binds with miR-485-3p to modulate the downstream target SRY-box 7 (SOX7). Silencing XIST impaired angiogenesis induced by hypoxia by modulating the miR-485-3p/SOX7 axis and suppressing the VEGF signaling pathway. These findings suggest that XIST, through the miR-485-3p/SOX7 axis, plays a crucial role in maintaining angiogenesis after hypoxia [132].

Yan et al. demonstrated that certain lncRNAs can promote angiogenesis. Their study revealed that lncRNA MACC1-AS1 attenuates microvascular endothelial cell injury and promotes angiogenesis by modulating the miR-6867-5p/TWIST1 axis in an in vitro model. The overexpression of MACC1-AS1 reduced cell apoptosis and oxidative stress, promoting cell proliferation, migration, and angiogenesis. Thus, lncRNA MACC1-AS1 holds potential for emerging therapies in IS by modulating the miR-6867-5p/TWIST1 axis [133].

Xu et al. observed a direct link between lncRNA D63785 and miR-422a, with their levels found to be correlated. Specifically, OGD/R conditions led to the downregulation of lncRNA D63785 due to increased m6A methylation, resulting in an increase in miR-422a and subsequent neuronal apoptosis. Conversely, silencing through lentiviral methyl-transferase-like protein 3 (METTL3) shRNAs reversed both the OGD/R-induced m6A methylation of lnc-D63785 and the increase in miR-422a, ultimately reducing neuronal cell death [134] (Table 3 and Figure 2).

These findings indicate that lncRNAs play a pivotal role in neuronal protection against hypoxia/anoxia that should be deeply understood further, in order to shed light on the concrete clinical use of these nucleic acids as promising therapeutic targets for treating cerebral ischemic conditions. Further exploration of the specific interactions between lncRNAs, miRNAs, and the involved signaling pathways could provide new therapeutic opportunities for enhancing neuronal repair and regeneration following ischemic stroke.

### 4.13. Experimental Studies That Reported the Effect on lncRNAs Modulation in Hypoxic/Anoxic Conditions Induced by Natural or Chemical Compounds

Hypoxic/anoxic conditions, oxidative stress, inflammation, and BBB disruption are significant events involved in the molecular and cellular damage in ischemic injury. Therefore, it is necessary to discover and test new therapeutic interventions capable of recovering and improving neuronal survival, thus preventing further CNS injuries [48,135].

In recent years, numerous in vivo and in vitro studies have investigated the effects of natural and chemical agents with neuroprotective proprieties and low toxicity, which exert their effects through the modulation of multiple signaling pathways, including the ones involving lncRNAs [136].

Several compounds, including berberine, a natural isoquinoline alkaloid capable of crossing the BBB, have been tested for their neuroprotective and anti-inflammatory effects against neurological disorders such as IS. In order to elucidate the biological effects of berberine, Song et. employed bioinformatics methodologies to unveil its potential targets and their involvement and relations with lncRNAs,. By matching the lncRNAs related to IS with those related berberine, they identified four potential target genes for berberine, including H19, HOTAIR, CASC2, and LINC00943, all involved in the treatment of IS. Particularly noteworthy is the high expression of H19 lncRNA in IS patients, suggesting its crucial regulatory role among the lncRNAs regulated by berberine. A software analysis revealed 20 key targets related to signaling pathways, including the MAPK, TLR, prolactin, TNF, and HIF-1 signaling pathways, which are associated with inflammation, immunity, and oxidative stress. Interestingly, a molecular docking analysis confirmed the strong binding capacity between berberine and these key proteins. It was also found that H19 lncRNA could directly be involved in the JNK1/c-Jun signaling pathway and EGFR one. In vitro experiments confirmed that berberine (10, 20, and 50 μM) restored the expressions of H19 lncRNA, p-JNK1/JNK1, p-c-Jun/c-Jun, and EGFR, which were significantly increased in SH-SY5Y cells cultivated under hypoxic conditions (1% O2) [137].

Other research groups such as Xu et al. have investigated the molecular mechanism by which melatonin (MT) improves delayed brain injury (DBI) following subarachnoid hemorrhage (SAH). MT administration improved DBI through the modulation of the H19/miR-675/HIF1A/TLR4 signaling pathways, leading to reduced apoptosis, inflammation, and neurobehavioral deficits in C57BL/6 J mice induced by an endovascular perforation model. Interestingly, MT enhanced H19 expression and miR-675 levels. Additionally, HIF-1α was identified as a target of miR-675, leading to the expression of TLR4 and the release of pro-inflammatory cytokines such as TNF-α. The study revealed a novel target that is the H19/miR-675/HIF1A/TLR4 signaling pathways, which results in being potentially useful in the management of brain damage. As a consequence, compounds such as MT, by modulating H19/miR-675/HIF1A/TLR4, could exert neuroprotective effects on post-SAH DBI [138].

Li et al. investigated the regulatory mechanism by which nicotine exposure modulates changes in lncRNA expression levels during neonatal brain hypoxic-ischemic development. They studied the effects of nicotine at a concentration of 102 mg/mL subcutaneously infused via osmotic mini-pumps in pregnant SD rats, and male pups where the right common carotid artery was incised and led to hypoxia. The authors demonstrated that nicotine exposure reduced H19 lncRNA expression and increased miR-181a levels, which, in turn, attenuated autophagy-related protein 5 (ATG5) and possibly led to the development of a brain phenotype sensitive to neonatal hypoxic/ischemic injury. Additionally, blocking miR-181a normalized ATG5 expression and reversed H/I-induced by perinatal nicotine exposure. H19 silencing enhanced miR-181a expression and exacerbated H/I-induced neonatal brain injury in the rat pups. In summary, the study identified the H19/miR-181a/ATG5 axis as a possible target to modulate the programming of the development of hypoxic-ischemic injury in neonatal brains [139].

Qiao et al. evaluated the role of rhabdomyosarcoma 2-associated transcript (Rmst), an lncRNA involved in neurogenesis, and the effects of the standardized *Ginkgo biloba* extract (EGb761) in OGD-induced bEnd.3 cells. The authors observed an increase in Rmst levels and a reduction in miR-150 expression in this cellular model after OGD exposure, indicating that Rmst overexpression was related to OGD-induced injury in the bEnd.3 cells. Further evidence on the Rmst role was reported in non-OGD-induced bEnd.3 cells. In this regard, Rmst silencing (without OGD exposure) exerted poor effects on cell proliferation, migration, and apoptosis in this in vitro model. Therefore, data suggested that Rmst can influence the bEnd.3 cell phenotypes acting on proliferation, migration, and apoptosis when they underwent OGD conditions. Moreover, as previously reported, miR-150 can target VEGF to modulate angiogenesis after IS. Since miR-150 is a target of Rmst, this could be useful for explaining the important role of Rmst in OGD-exposed bEnd.3 cells. Consequently, it was shown that, EGb761 100 μg/mL treatment, by modulating Rmst and, indirectly, miR-150 levels, exerted a neuroprotective role in OGD bEnd.3 cells. In conclusion, EGb761 modulating the Rmst/miR-150 axis can be a useful compound for reducing the brain damage induced by hypoxia–ischemia in microvascular endothelial cells [140].

Several phytochemicals possess wide pharmacological properties and they could be effectively used to treat many diseases.

In light of these statements, many research groups have investigated the role of phytocompounds in the field of these specific injuries.

For instance, Yang et al. investigated the effect of β-Asarone on PC12 cells induced by hypoxia using cobalt chloride (CoCl_2_) to create the model. They noted a high level of the lncRNA ribonuclease P RNA component H1 (RPPH1) in the PC12 cells. This lncRNA plays a role in the RPPH1/miR-542-3p/DEDD2 axis, where it acts as sponge for miR-542-3p. In turn, this miRNA influences the level of death effector domain containing 2 (DEDD2), exerting a role in exacerbating the pathological state. The study demonstrated that β-Asarone protects PC12 cells against hypoxia-induced injury by suppressing theRPPH1/miR-542-3p/DEDD2 axis [141].

Wang et al. studied the DEX effect on hippocampal neuronal cells, demonstrating that DEX exerted a neuroprotective action by regulating the SNHG16/miR-10b-5p/BDNF axis. The authors aimed to elucidate the role of lncRNA SNHG16 in DEX-induced brain protection using rats which underwent MCAO surgery, and OGD-treated HT22 hippocampal neurons, in order to investigate the underlying molecular mechanisms. Through a luciferase report assay, they revealed the interactions between SNHG16 and miR-10b-5p, as well as between miR-10b-5p and the BDNF gene. They demonstrated that DEX inhibited miR-10b-5p expression by acting as a sponge, leading to an increase in SNHG16 and BDNF levels in a dose-dependent manner. Through this mechanism, the study showed that DEX attenuated neurological damage and increased the cell viability in the neurons. These results suggest that DEX could serve as a neuroprotective factor against ischemia-anoxia-mediated brain injury [136].

Another interesting compound is Gastrodin (GAS), which is a traditional Chinese medicine ingredient used in the treatment of vascular and neurological diseases. In order to understand more of its pharmacological properties and mechanisms, Zhang et al. investigated the effects of GAS on cerebral I/R injury using MCAO rats and OGD/R rat primary cortical neurons as research models. MCAO treatment increased the expressions of IL-1β, IL-18, cleaved caspase-1, and NLRP3. However, these protein expressions decreased in rats pretreated with GAS (7 days before I/R surgery) and treated (7 days after I/R surgery). The results indicate that GAS reduced I/R-induced inflammation in neuronal cells through the lncRNA NEAT1/miR-22-3p axis [142].

Hu et al. reported the effects of Propofol (PPF), a hypnotic drug used as sedative agent, on CI/RI. They used an MCAO/R mouse model and PC12 cells exposed to H/R as an in vitro CI/RI model. The aim of this study was to find the cellular mechanism that explains the positive effect of PPF on this pathological condition. They detected the expressions of lncRNA MALAT1 and miR182-5p using qRT-PCR. The results suggested that the increase in MALAT1 level, in turn, increased the expression of TLR4 by suppressing miR-182-5p. Overall, the study result demonstrated that PPF protects brain tissues by regulating the MALAT1/miR-182-5p/TLR4 axis [143] (Table 4).

In conclusion, lncRNAs play roles in the pathological mechanisms associated with cerebral ischemia. Several lncRNAs, including H19, Rmst, and NEAT1, have been identified to be involved in inflammation, oxidative stress, and cell death processes under hypoxic/anoxic conditions. Moreover, various substances such as berberine, melatonin, DEX, gastrodin, and propofol appear to exert neuroprotective effects by modulating these specific lncRNAs and their associated signaling pathways.

### 4.14. Experimental Studies That Have Focused on the Change in the Expression of Multiple lncRNAs in Hypoxic/Anoxic Conditions

Hypoxic/anoxic conditions lead to alterations in the transcriptomic profiles of several LncRNAs [36], as explained throughout our own research. For instance, Wang et al. identified 106 upregulated lncRNAs and 104 downregulated lncRNAs. Through GO and KEGG pathway analyses, the research team identified cellular apoptosis, natural killer cell-mediated cytotoxicity, and autophagy as the most involved pathways [144].

Furthermore, Wang et al. identified 33 significantly dysregulated lncRNAs. Among the differentially expressed lncRNAs, the 32856 gene (Gm32856), small nucleolar RNA host gene 17 (snhg17), G protein-coupled receptor 137b-pseudogene (Gpr137b-ps), subunit 6a (Cct6a), and chaperonin-containing Tcp1 were found to be overexpressed. It is noteworthy that lncRNA Gpr137b-ps modulates angiogenesis processes and its potential targets include LOX1, STAT3, and VEGF. Therefore, the study revealed that lncRNA Gpr137b-ps could be potential candidate to promote angiogenesis after I/R [145].

The study of Zhao et al. suggests TNFRSF17 as a target for the prognosis, diagnosis, and treatment of HIBD, because they showed that this lncRNA overexpressed reduces the apoptotic rate; instead, TNFRSF17 silencing led to a high rate of apoptosis under hypoxia. This article showed that around 617 lncRNA transcripts showing aberrant expression were detected in the hippocampal tissues of neonatal rats 24 h after HIBD [146]. He et al. conducted a comprehensive analysis of the transcriptomic profiles in the mouse hippocampal neuron HT22 cell line under NMDAR knockdown and NMDAR knockdown with H/R injury conditions to explore their effects. Three experimental groups were utilized: si-NMDAR:si-NC, si-NMDAR+H/R:si-NC, and si-NMDAR+H/R:si-NMDAR. Significant differential expressions of 101, 58, and 96 lncRNAs were observed in each comparison. The primary aim of this study was to characterize the expression profiles of proteins, mRNAs, and lncRNAs in neurons following NMDAR knockdown and H/R injury. The results revealed that, among all the differentially expressed lncRNAs, XLOC_161072 and XLOC_065271 were significantly upregulated after NMDAR knockdown with H/R compared to only NMDAR knockdown. On the other hand, the lncRNAs XLOC_159404 and XLOC_031922 were significantly downregulated after NMDAR knockdown with H/R, compared to only NMDAR knockdown, showing that some lncRNAs indeed respond to hypoxic stimulation [147].

Ewida et al. focused their attention on the lncRNA HIF1A-AS2 and lncRNA LINK-A. Using qRT-PCR, the study evaluated the transcriptional levels of lncRNAs in the serum of stroke patients. The results showed the upregulations of HIF1A-AS2 and HIF1-α transcript, along with a downregulation of lncRNA LINK-A [148].

Zhou et al. evaluated the expression profiles of lncRNAs and mRNAs in the brains of HIBD in neonatal rats using next-generation high-throughput RNA sequencing. The study results identified a total of 328 differentially expressed lncRNAs and 7157 differentially expressed mRNAs. Additionally, with the aim of discovering new pathways and biological processes involved in neonatal HIBD, the authors analyzed the relationships between the lncRNAs and their target mRNAs. An IKEGG and gene ontology analysis reported a link between lncRNAs and several signaling pathways, including Janus tyrosine kinase-signal transducer and activator of transcription (JAK-STAT), NF-κB, Toll-like receptor (TLR), Notch, and MAPK. Consequently, the study results demonstrated that the identification and characterization of lncRNAs can be valuable for therapeutic research in neonatal HIBD [149].

All these studies collectively suggest that hypoxic/anoxic conditions are able to enhance the molecular response mediated by lncRNAs, opening the door to the future possibility of utilizing them as therapeutical target (Table 5).

## 5. Studies about CircRNAs in Hypoxic/Anoxic Conditions

We identified studies aimed at analyzing transcriptomic profiles and their changes in models compared to controls, as well as papers focusing on specific circRNAs that can either be neuroprotective or exacerbate hypoxic/anoxic conditions. Moreover, we identified studies demonstrating the existence of substances with the ability to influence the underlying mechanisms involving circRNAs.

The miRNA sponging ability of circRNAs is well-known. A study performed by Wei et al. focused on the RNA expression profile to identify the key miRNA–circRNA interaction networks in HIE. Through a bioinformatics analysis of two RNA-seq datasets and the corresponding metadata acquired from the Genes Expression Omnibus (GEO) database (GSE164727 and GSE121178), the research group identified 5512 differentially expressed coding and non-coding RNAs, out of which 2432 were circRNAs. Particularly, the article suggested further research on hsa_circ_0045698 and hsa_circ_0068397 due to their high expression levels. These circRNAs were identified as being central in HIE, indicating a potential role of circRNAs HIE development through circRNA/miRNA interactions [150].

In an RNA-seq analysis of mouse brains following HIBD, Jiang et al. identified 66 differentially expressed circRNAs (20 upregulated and 46 downregulated). To validate these findings, the research group performed a complementary experiment using RT-qPCR, analyzing four circRNAs that showed differential expressions in the initial RNA-seq data. The results were consistent [151].

Instead, Dong et al. studied circRNA expression in the peripheral blood of neonates with HIE using circRNA microarray profiling, and identified 456 significantly differentially expressed circRNAs (250 upregulated and 206 downregulated). This study suggested a potential relationship between circRNAs and HIE in neonates [152].

In a 2019 paper, the research team Ma et al. analyzed the neutrophil transcriptome of five asymptomatic Moyamoya disease (MMD) patients. MMD is a pathology linked to angiogenesis, caused by the progressive stenosis of the cerebral arteries of the brain base. This research discovered 123 circRNAs expressed differentially (54 upregulated and 69 downregulated compared to controls) from analyzing the profile of 13,439 circRNAs through a microarray. This study suggests that angiogenesis is involved in asymptomatic MMD [153].

The research by Wu et al. aimed to evaluate circRNAs as biomarkers for detecting blood inflammation in people with unruptured intracranial aneurysms (UIAs) accompanied by an abnormal wall enhancement (AWE) observed through high-resolution vessel wall imaging (HR-VWI). This study found 412 differentially expressed circRNAs between UIA patients and healthy controls and 231 UIA patients with AWE and without AWE. These circRNAs could be novel inflammatory biomarkers for assessing UIA patients. Particularly, they focused on Hsa_circ_0007990 due to its higher fold-change compared to other differentially expressed circRNAs, suggesting further investigation [154].

In a study of Yang et al., patients with acute IS showed a significant reduction in circFOXP1 levels compared to healthy individuals. The authors suggested an inverse correlation between circFOXP1 expression and IS prognosis. Specifically, circFOXP1 reduced apoptosis by affecting the Bax and caspase-3 expression in A172 cells after OGD/R treatment. These findings were confirmed in vivo by using adeno-associated virus to overexpress circFOXP1 in mice subjected to transient MCAO as a brain ischemic model. The overexpression of circFOXP1 improved neurological functions, enhanced functional recovery, and reduced brain damage. Moreover, circFOXP1 binding to STAT3 led to a decrease in its ubiquitination and degradation, resulting in reduced apoptotic signaling after hypoxia [155].

From these studies, it can be concluded that hypoxic conditions significantly influence circRNA expression, suggesting that circRNAs may be implicated in the response to hypoxic brain damage. Although the functional roles of circRNAs in hypoxic brain injury are not fully understood, some studies have investigated specific circRNAs, as in the last article.

Cai et al. focused on circARF3 and studied its role in regulating BBB injury in subarachnoid hemorrhage (SAH) rats and hypoxia-induced vascular endothelial cell (VEC) injury in vitro. They demonstrated that miR-31-5p has a pro-inflammatory effect, and the overexpression of circARF3 reduced hypoxia-induced damage and neuroinflammation involving the miR-31-5p/MyD88/NF-κB axis [156].

On the other hand, Sun et al. focused on Circ_0090002 using HBMEC after OGD. This circRNA modulated HECTD1 expression via miR-186-5p, enhancing viability, migration, apoptosis, and oxidative stress. So, circ_0090002 could be a potential target for the treatment of cerebral ischemic injury [157].

Another study by Ren et al. highlighted the role of circ-RNA Memo1 in a model of cerebral H/R, using HBMVECs. They found that Memo1 silencing, already known for its upregulation in other ischemic models, positively affected the H/R conditions in the aforementioned model by inhibiting the activation of the ERK/NF-κB signaling pathway. This silencing led to a reduction in oxidative stress, inflammatory response, and cell apoptosis. Based on this evidence, Memo-1 is indeed a potential target for Cerebral H/R treatment [158].

Using the same model, Yang et al. noted lower levels of circPHKA2 after OGD and in the blood of acute ischemic stroke (AIS) patients. Consequently, they focused on this circRNA and demonstrated that its overexpression attenuated the OGD-induced inhibition of cell proliferation and increased the migration of HBMEC. CircPHKA2 particularly reduced cell apoptosis, ER stress, and oxidative stress. The authors suggest that circPHKA2 might suppress OGD-induced effects by modulating SOD2 expression through miR-574-5p [159].

On the other hand, Jiang et al. showed that circRNA ANRIL contributed to cell damage in OGD/R-treated HBMECs by promoting apoptosis and inflammatory response through the NF-κB pathway via sponging miR-622, leading to an increase in the secretion of IL-1β, IL-6, TNF-α, and MCP-1 [160].

Similar to circANRIL, CircFUNDC1 expression increased, as demonstrated by Bai et al. In fact, circFUNDC1 knockdown recovers the OGD-weakened cell viability, survival, migration, and angiogenesis of HBMECs by targeting the miR-375/PTEN axis. Moreover, the authors showed that this circRNA increased in the peripheral blood of IS patients. Based on these results, this article suggests that the circFUNDC1 cloud is a biomarker for predicting IS [161].

Cao et al. investigated the role of ciRNA cZNF292 in rat NSCs to mimic the pathological conditions of HIE. Using OGD/R stress injury on NSCs and both inhibiting or overexpressing cZNF292, they found that cZNF292 silencing alleviated OGD/R-induced injury via the upregulation of miR-22 [162].

In a recent study, Feng et al. investigated the role of circDLGAP4-carrying exosomes derived from human umbilical cord mesenchymal stem cells in the CI/RI angiogenesis of HBMECs and in an I/R rat model. They proposed that exosomal circDLGAP4 promoted cell viability, migration, and proliferation in HBMECs by targeting miR-320 and activating the KLF5/PI3K/AKT pathway. Moreover, exosomal circDLGAP4 derived from umbilical cord mesenchymal stem cells alleviated cerebrovascular injury in an I/R rat model using the same pathway. These data suggest that this circRNA could be a potential key factor in CI/RI treatment [163].

We also identified articles in which the authors used substances influencing circRNA expressions. The research group led by Zhang et al. discovered a total of 23 differentially expressed circRNAs in mice with high-altitude hypoxia-induced brain injury and mice treated with *Gymnadenia conopsea* (L.) R. Br. compared to normal controls. The genus *Gymnadenia* R.Br. is a perennial herb belonging to the family Orchidaceae and it has pharmacological activities against gastric ulcers, immunoregulation, anti-hyperlipidemia, anti-anaphylaxis, anti-silicosis, anti-cancer, and neuroprotective properties [164]. The authors focused on the roles of these circRNAs through a KEGG analysis. The circRNAs were found to be involved in the following pathways: the TNF signaling pathway, apoptosis, neurotrophin signaling, and the MAPK signaling pathway. As a result, they proposed them as potential biomarkers [165].

Wang et al. studied the neuroprotective effects of DEX on hippocampal neurons under H/R treatment. DEX is an isomer of medetomidine. It is a highly selective α2-adrenoceptor agonist in the central nervous system, resulting in a sympatholytic effect [166]. This article demonstrated that DEX relieved the H/R-induced apoptosis and inflammatory responses in HT-22 cells via the circ-CDR1as/miR-28-3p/TRAF3 cascade. In fact, upregulated circ-CDR1as reversed the protective effects of DEX [167] (Table 6 and Figure 3).

These studies demonstrated the involvement of circRNAs in cerebral hypoxia and ischemic injuries. Transcriptomic analyses revealed distinct circRNA expression patterns under hypoxic stress. Specific circRNAs, including circFOXP1, circARF3, circ_0090002, circMemo1, circPHKA2, CircANRIL, CircFUNDC1, cZNF292, CircDLGAP4, and CircCDR1as, were identified as being relevant in these processes. Certain substances, such as DEX and *Gymnadenia conopsea* (L.) R. Br., were found to influence circRNA expression, affecting inflammatory and apoptotic responses. These findings offer potential for targeted therapeutic approaches in neurological disorders like cerebral ischemia.

## 6. Conclusions

LncRNAs and circRNAs are abundantly expressed in brain tissue. Since there are few studies on circRNAs, it is necessary investigate their involvement in anoxic/hypoxic brain damage more.Transcriptomic analyses revealed the involvement of circRNAs such as circFOXP1, circARF3, circ_0090002, circMemo1, circPHKA2, CircANRIL, CircFUNDC1, cZNF292, CircDLGAP4, and CircCDR1as in various processes, such as inflammation and apoptotic response. LncRNAs are crucially significant and potentially useful for the identification and management of brain damage in anoxic/hypoxic conditions. H19, MALAT1, PVT1, and MIAT are involved in the modulation of signaling pathways related to apoptosis, autophagy, inflammation, proliferation, cell survival, cell growth, and angiogenesis, as it has been shown throughout our review. Instead, the silencing of GAS5, SNHG14, MEG3, and BDNF-AS leads to a reduction in apoptosis and oxidative stress. In contrast, the overexpression of SNHG1, SNHG3, PEG13, and SNHG16 improved cell survival and reduced apoptosis. LncRNAs could be potential candidates as biomarkers for cerebral ischemic events, as well as potential therapeutic targets to counteract brain damage associated with anoxic/hypoxic conditions. Despite the findings highlighted by the available data, there are still few studies on circRNAs; hence, further investigation into their involvement in anoxic/hypoxic brain damage is necessary.

## Figures and Tables

**Table 1:** Studies that summarized the involvement of lncRNAs in hypoxic/anoxic conditions.

***Studies that Summarized the Involvement of LncRNAs SNHG Family in Hypoxic/Anoxic Conditions***			
SH-SY5Y OGD/R-induced	SNHG1	The overexpression of SNHG1 or the KO of miR-140-5p alleviated hypoxia-induced damage. Overall, the results proved that lncRNA SNHG1 could be novel therapeutic target in hypoxic brain injury	[63]
Primary BMEC mice OGD/R-induced	SNHG1	Snhg1 silencing led to worsening the BMEC apoptosis that was OGD/R-induced, so confirmed the protective effect of lncRNA Snhg1in in IS model	[64]
C57Bl/6 mice with ligation of the right internal carotid artery. Primary hippocampal cell culture with shRNA or miRNA mimic transfection	SNHG3	Snhg3 silencing led to reduction in cell viability and promoted the apoptosis, in vivo Snhg3 overexpression improved brain neurological function in the animals and reduced HIBD	[65]
OGD/R-induced primary neurons	SNHG12	SNHG12 knockdown exacerbated apoptosis induced by OGD/R	[66]
HT22 cells of I/R model	SNHG12	SNHG12 increased cell activity and inhibited oxidative stress through inhibition of SIRT1/FOXO3a signaling-mediated autophagy	[67]
N2A cells ODG/R-induced MCAO/R mice	SNHG14	SNHG14 silencing led to an increase in miR-98-5p and BCL2L13 depletion. So, SNHG14 is beneficial to OGD/R	[68]
MCAO male SD rats OGD-induced primary cortical neurons of SD embryos rat	SNHG14	SNHG14 silencing led to a reduction in the infarct volume and improved neurological deficits in MCAO rats	[69]
H/I mice model PC12 cells OGD-stimulated	SNHG15	SNHG15 suppression alleviated H/I brain injury by modulating the miR-153-3p/SETD7 axis	[70]
HBMECs OGD/R-induced	SNHG16	The overexpression of SNHG16 led to a decrease in miR-15a-5p and increased the Bcl-2 expression levels, as well as reduced apoptosis and enhanced cell survival	[71]
***Studies that Summarized the Involvement of LncRNA MALAT1 In Hypoxic/Anoxic Conditions***			
C57BL/6 mice with right common carotid artery occlusion C57BL/6 mice hippocampal neurons with H/I	MALAT1	MALAT1 acting as a miR-429 sponge led to an increment in WNT1 expression promoting apoptosis, as shown by expression levels of cleaved caspase-3 and the ratio of Bax to Bcl-2 both in vitro and vivo	[72]
HBMECs OGD-induced	MALAT1	Through sponging miR-126, MALAT1 inhibited proliferation and induced apoptosis of OGD-induced HBMECs	[73]
bEnd.3 OGD-induced	MALAT1	The MALAT1-miR-200c-3p-SIRT1 pathway could be interesting to modulate for influencing autophagy and cell survival for neuroprotection	[74]
BMECs of C57BL/6J mice OGD/R-induced	MALAT1	MALAT1 increased autophagy and cell survival in BMECs	[75]
MCAO mice BMECs OGD-induced	MALAT1	MALAT1 interactions reduced cell death and inflammation	[76]
MCAO C57BL/6J mice BMECs OGD/R-induced	MALAT1	Following OGD/R, MALAT1 regulated angiogenesis	[77]
***Studies that Summarized the Involvement of LncRNA H19 In Hypoxic/Anoxic Conditions***			
Neonatal HIE SD rats	H19	H19 overexpression reduced both the nervous damage and autophagy of brain tissue, as well as improved neurological functions	[78]
Primary neuron cells rat OGD-induced HIBD SD rat induced by partial occlusion of carotid artery	H19	HIBD can be prevented by overexpression of the lncRNA H19	[79]
HIBD neonatal rats	H19	H19 overexpression suppressed apoptosis in rat cardiomyocytes	[80]
Blood samples of IS patients MCAO/R rats SH-SY5Y cells OGD-induced	H19	Hypoxia/ischemia-induced neuronal injury was attenuated by blocking the lncRNA H19-miR-19a-Id2 axis	[81]
PC-12 cells induced by hypoxia	H19	Overexpression of lncRNA H19 led to an increase in cell damage induced by hypoxic conditions	[82]
IS patients MCAO mouse BV2 cell OGD-induced	H19	H19 could be a useful marker as well as a potential therapeutic target for IS patients	[83]
***Studies that Summarized the Involvement of LncRNA GAS5 in Hypoxic/Anoxic Conditions***			
Blood of CA/CCR patients OGD/R-inducted astrocytes	GAS5	Inhibiting PI3K/Akt signaling through INPP4B, lncRNAGAS5/miR-137 is a hypoxia-responsive axis involved in astrocyte–microglia crosstalk.	[84]
HIBD neonatal rat model established and treated with shRNA-GAS5 or antagomir miR-128-3p	GAS5	Through the microRNA-128-3p/Bax/Akt/GSK-3β axis, downregulation of GAS5 prevents mitochondrial apoptosis and HIBD in neonatal rats	[85]
Primary rat cortical cell hypoxia-treated B35 cell with hypoxic treated MCAO SD rats	GAS5	GAS5 can lead to worsening of cell apoptotic processes in hypoxic conditions targeting miR-221/PUMA axis. Consequently, GAS5 could be useful in ischemic stroke treatment	[86]
***Studies that Summarized the Involvement of LncRNA NEAT1 in Hypoxic/Anoxic Conditions***			
BMECs OGD-induced	NEAT1	NEAT1 facilitates survival and angiogenesis in OGD-induced BMECs via targeting miR-377	[87]
Neonatal HIBD mouse model was established via right common carotid artery occlusion	NEAT1	The findings demonstrated that NEAT1 alleviated HIBD in mice by binding to miR-339-5p	[88]
***Studies that Summarized the Involvement of LncRNA MIAT in Hypoxic/Anoxic Conditions***			
Neuro2A cells OGD-induced Neonatal rat with permanent unilateral carotid ligation	MIAT	The overexpression of MIAT reduced neuron apoptosis and relieved HI injury of neonatal rats through miR-211/GDNF	[89]
MCAO SD rats Primary rat neurons OGD/R- stimulated	MIAT	MIAT exacerbated I/R injury by disrupting redox homeostasis in neurons	[90]
***Studies that Summarized the Involvement of LncRNA PVT1 in Hypoxic/Anoxic Conditions***			
MCAO mice Primary neurons OGD/R-induced	PVT1	PVT1 silencing led to a reduction in infarct volume and improved neurological behavior in MCAO mice	[91]
HUVECs OGD-induced	PVT1	PVT1 alleviated hypoxia-induced endothelial apoptosis by enhancing autophagy through the miR-15b-5p/ATG14 and miR-424-5p/ATG14 pathways	[92]
***Studies that Summarized the Involvement of LncRNA Peg13 in Hypoxic/Anoxic Conditions***			
Neonatal HIBD mice Mouse hippocampal neurons OGD-induced	PEG13	Peg13 exerted an anti-apoptotic role, acting as a sponge for miR-20a-5p targeting XIAP	[93]
bEnd.3 cells OGD/R-induced MCAO mice	PEG13	Peg13 alleviated I/R-induced neurological deficit, cerebral infarct, and cerebral edema	[94]
***Studies that Summarized the Involvement of LncRNA MEG3 in Hypoxic/Anoxic Conditions***			
PC12 cells induced by hypoxia	MEG3	MEG3 silencing performed by siRNA played a neuroprotective function, preventing hypoxia-induced injury in PC12 via the modulation of protein involved in apoptosis and cell proliferation	[95]
MCAO C57BL/6J mice	MEG3	Silencing MEG3 resulted in being protective against I/R-induced ischemic damage in vivo and improved overall neurological functions	[96]
PC12 cells cultivated in hypoxic conditions	MEG3	MEG3 led to an increase in hypoxic damage in PC12 cells by targeting miR147 and the downstream target Sox2	[97]
260 neonatal female or male C57/BL6 mice with right cervical vessels ligated with a double bandage	MEG3	MEG3 silencing improved the therapeutic effect of DEX on HIBD in neonatal mice through the expression of miR-129-5p	[98]
***Studies that Summarized the Involvement of LncRNA BDNF-AS in Hypoxic/Anoxic Conditions***			
Mice received ligation of the unilateral common carotid artery Primary hippocampal neuron with hypoxic and oxidative stress induced	BDNF-AS	Silenced BDNF-AS lncRNA attenuated HI events	[99]
HCN2 and human astrocytes after H/R	BDNF-AS	lncRNA BDNF-AS knockdown suppressed apoptosis	[100]

OGD/R: oxygen-glucose deprivation/reperfusion; KO: knockout; BMEC: brain microvascular endothelial cells; IS: ischemic stroke; HIBD: hypoxic ischemic brain damage; I/R: ischemic/reperfusion; MCAO/R: middle cerebral artery occlusion/reperfusion; MCAO: middle cerebral artery occlusion; SD: Sprague Dawley; H/I: hypoxic ischemic; HBMECs: human brain microvascular endothelial cells; Bcl-2: B-cell lymphoma 2; MALAT1: Metastasis-Associated Lung Adenocarcinoma Transcript 1; bEnd.3: brain microvascular endothelial cells; OGD: oxygen-glucose deprivation; HIE: hypoxic-ischemic encephalopathy; CA/CCR: Cardiac Arrest/Cardiopulmonary Cerebral Resuscitation; GAS5: Growth Arrest-Specific 5; PUMA: p53-upregulated modulator of apoptosis; NEAT1: Nuclear paraspeckle assembly transcript 1; MIAT: myocardial Infarction Associated Transcript; PVT1: plasmacytoma variant translocation 1; HUVECs: human umbilical vein endothelial cells; ATG14: autophagy-related 14; XIAP: X chromosome-linked inhibitor of apoptosis; MEG3: Maternally expressed gene 3; DEX: dexmedetomidine; and HCN2: human cortical neurons.

**Table 2:** Experimental studies about other lncRNAs that play a role in exacerbating hypoxic/anoxic conditions.

Models	LncRNAs Involved	Conclusions	Ref.
Neuronal cells OGD-induced Blood of 44 IS patients and 37 healthy controls	LOC105376244	LOC105376244 results in being dysregulated in IS patients and it is in turn regulated by miR-145–EPHA4 interaction	[113]
Neuronal cells OGD/R-stimulated Mouse brains subjected to transient focal ischemia	AK038897	AK038897 plays a role in cerebral I/R injury	[114]
PC12 cells OGD/R-stimulated Plasma of patients with cerebral ischemia reperfusion	KCNQ1OT1	Upregulated KCNQ1OT1 aggravates cerebral I/R injury	[115]
Plasma of neonatal HIE patients hBMVECs OGD/R-induced	HOTAIR	LncRNA HOTAIR modulating EZH2, led to apoptosis of hBMVECs	[116]
HIBD rat model Primary cortical neurons of rats OGD-stimulated	CRNDE	The silencing of CRNDE alleviated brain injury after HI and inhibited neuronal apoptosis, both by promoting autophagy	[117]
H/R SH-SY5Y cells MCAO rat model	PINK1-AS	PINK1-AS/miR-203/ATF2 axis is involved in NOX2 expression regulation	[118]
N2a cells OGD-stimulated	Gm11974	Gm11974 suppression provides protection against cerebral I/R injury, suggesting that this protective effect is mediated by the miR-766-3p/NR3C2 axis	[119]
N2a cells OGD/R-exposed	RMST	RMST decrement causes the abrogation of the OGD/R-triggered apoptosis and oxidative stress	[120]
HIE neonatal SD male rats	Vof-16	Vof-16 KO improves neurological damage as well as spatial learning and memory functions in HIE rats	[121]
MCAO mice BV-2 cells were exposed to high glucose followed by H/R	Fendrr	Fendrr is able to protect against the ubiquitination and degradation of NLRC4 protein through E3 ubiquitin ligase HERC2, thereby accelerating the pyroptosis of microglia	[122]

OGD: oxygen-glucose deprivation; IS: ischemic stroke; OGD/R: oxygen-glucose deprivation/reperfusion; I/R: ischemic/reperfusion; HIE: hypoxic ischemic encephalopathy; HBMVECs: human brain microvascular endothelial cells; HIBD: hypoxic ischemic brain damage; CRNDE: colorectal neoplasia differentially expressed; H/I: hypoxic ischemic; H/R: hypoxia/reoxygenation; MCAO: middle cerebral artery occlusion; KO: knockout; and BDNF-AS:BDNF Antisense RNA.

**Table 3:** Experimental studies about other lncRNAs possessing neuroprotective properties.

Models	LncRNAs Involved	Conclusions	Ref.
MCAO mice Microglial cell OGD-induced	1810034E14Rik	The overexpression of lncRNA1810034E14Rik leads to a reduction in inflammatory cytokine levels and brain damage	[123]
OGD/R-induced HBMVECs	FAL1	Overexpressed FAL1 reduces inflammation	[124]
MCAO mice OGD/R-induced HBMECs	ROR	Upregulation in the expression of ROR inhibits the activation of TNF-α/P-ASK-1-mediated apoptosis, which ultimately alleviates hypoxia in brain tissue	[125]
HIE SD neonatal rats	Vi4	LncRNA Vi4, by miR-185-5p modulation, leads to an improvement in motor functions, learning. and memory deficits	[126]
HIE whole blood (8 infants) OGD/R-induced SH-SY5Y	LINC00938	LINC00938 contributes to the promotion of neuronal cell survival. It achieves this by inhibiting the activation of p-JNK and p-p38 MAPK signaling pathways	[127]
HIE neonate rats	TCONS_00041002	TCONS_00041002 participates in the cell apoptosis and neuronal survival of HIE and represents a potential new target for the treatment of HIE	[128]
PC12 cells OGD-induced	ANRIL	ANRIL is able to reduce HIBD by targeting the miR-378b/ATG3 axis	[129]
Serum of stroke patients MCAO/R rats OGD/R-induced microglial cells	OIP5-AS1	lncRNA OIP5-AS upregulation exerts neuroprotective effects	[130]
MCAO C57BL/6 mice Primary cortical neurons OGD-induced	HOTTIP	Overexpression of HOTTIP leads to an increase in cell viability and reduces apoptosis, as well as increasing and improving glycolytic metabolism via the upregulation of HK-2 expression levels, leading to the reduction in OGD-induced neuronal injury	[131]
HBMECs induced by hypoxia	XIST	The study results suggest that XIST involving miR-485-3p/SOX7 axis exerts an important role in maintaining angiogenesis processes after hypoxia	[132]
HBMECs induced by hypoxia	MACC1-AS1	The study found that lncRNA MACC1-AS1, by modulating the miR-6867-5p/TWIST1 axis, could help to discover emerging therapies useful for IS	[133]
SH-SY5Y cells and primary murine neurons both OGD/R stimulated	D63785	The silencing of lnc-D63785 leads to an increase in Mir-422a, thus reducing apoptosis	[134]

MCAO: middle cerebral artery occlusion; OGD: oxygen-glucose deprivation; OGD/R: oxygen-glucose deprivation/reperfusion; HBMVECs: human brain microvascular endothelial cells; ROR: regulator of reprogramming; HIE: hypoxic ischemic encephalopathy; SD: Sprague Dawley; MAPK: mitogen activated protein kinase; HIBD: hypoxic ischemic brain damage; MCAO/R: middle cerebral artery occlusion/reperfusion; HOTTIP: HOXA transcript at the distal tip; HK-2: hexo-kinase 2; XIST:X-inactive specific transcript; SOX7:SRY-box 7; and IS: ischemic stroke.

**Table 4:** Summary of the in vitro and in vivo studies that showed the effects of natural or chemical compounds on lncRNA modulation in anoxic/hypoxic conditions.

Model	Compound	LncRNAs Involved	Results	Ref.
MCAO rats OGD-treated HT22 hippocampal neurons	Rats DEX (25 μmol/kg, 50 μmol/kg, 100 μmol/kg body weight) injected via external jugular vein and HT22 neuronal cells treated with DEX (25 μM, 50 μM, and 100 μM) for 24 h	SNHG16	DEX attenuates neurological damage, increasing neurons’ viability and promoting lncRNA SNHG16 and BDNF levels, as well as reducing miR-10b-5p expression	[136]
SH-SY5Y cells in hypoxia condition	Berberine at a doses of 10, 20, and 50 μM	H19, HOTAIR, CASC2 and LINC00943	Berberine treatment reduces IS in in vitro model, through inhibition of lncRNA H19/EGFR/JNK1/c-Jun axis	[137]
C57BL/6 J mice with endovascular perforation	MT at a dose of 150 mg/kg via intraperitoneally 12 h after surgery	H19	MT administration improves neurobehavioral deficits and reduces both apoptosis and inflammation levels, leading to an increase in H19 and miR-675, as well as a reduction in HIF-1α and TLR4	[138]
Pregnant SD rats and male pups incised the right common carotid artery and exposed to hypoxia	Nicotine at a dose of 102 mg/mL infused subcutaneously by osmotic mini-pumps	H19	Nicotine exposure leads to an increase in H19 and miR-181a, as well as a decrease in ATG5 and thus the possible onset of the brain phenotype sensitive to neonatal H/I, while miR-181a block induces a reduction in H/I induced by perinatal nicotine exposure	[139]
OGD-induced bEnd.3 cells	EGb761 at a dose of 100 μg/mL 2 h before OGD exposure	RMST	EGb761 pretreatment leads to apoptosis reduction as well as an increase in lncRNA RMST and miR-150	[140]
PC12 cells treated with CoCl_2_	β-Asarone (20, 30, and 45 μg/mL)	RPPH1	β-Asarone exerts neuroprotective effects, reducing hypoxia-induced damage, by suppressing RPPH1/miR-542-3p/DEDD2 axis	[141]
MCAO rats OGD/R rat primary cortical neurons	GAS 50 mg/kg	NEAT1	GAS attenuates I/R-induced inflammation in neuronal cells via lncRNA NEAT1/miR-22-3p axis	[142]
MCAO/R Kunming mice PC12 cells H/R-induced	PC12 cells pre-treated with PPF 50 μM 1 h before H/R induction and Rats infused intravenously with PPF (60 mg/kg, 10 min before MCAO	MALAT1	PPF exerts neuroprotective effects thanks to increments in MALAT1 and miR-182-5p, as well as a reduction in TLR4 expression	[143]

MCAO: middle cerebral artery occlusion; OGD: oxygen-glucose deprivation; DEX: dexmedetomidine; BDNF: Brain-derived neurotrophic factor; IS: ischemic stroke; EGFR: epidermal growth factor receptor; MT: melatonin; HIF-1α: hypoxia-inducible factor-1alpha; TLR4: Toll-like receptor 4; SD: Sprague Dawley; ATG5: autophagy-related protein 5; H/I: hypoxic ischemic; bEnd.3: brain microvascular endothelial cells; EGb761: Ginkgo biloba extract; RMST: rhabdomyosarcoma 2-associated transcript; CoCl_2_: cobalt chloride; RPPH1: ribonuclease P RNA component H1; DEDD2: death effector domain containing 2; MCAO/R: middle cerebral artery occlusion/reperfusion; GAS: Gastrodin; NEAT1: nuclear-enriched abundant transcript 1; I/R: ischemic/reperfusion; H/R: hypoxia/reoxygenation; PPF: Propofol; and MALAT1: Metastasis-associated lung adenocarcinoma transcript 1.

**Table 5:** Summary of the studies that through bioinformatics analysis reported changes in expression levels of lncRNAs in in anoxic/hypoxic conditions.

Models	LncRNAs Involved	Conclusions	Ref.
Neonatal rats with the right common carotid artery permanently ligated Primary OPC OGD-induced	↑106 ↓104	The study suggests the contribution of lncRNAs to the development of preterm WMI	[144]
BEnd.3 of mouse	↑18 ↓15	Multiple lncRNAs exhibit altered expression, with a focus on lncRNA Gpr137b-ps being a potential candidate to promote angiogenesis after I/R	[145]
Neonatal rats with the left common carotid artery ligated and transferred in a low-oxygen chamber	617 aberrantly expressed	HI injury alters the expression profiles of lncRNAs in neonatal rat brains	[146]
Mouse hippocampal neuron HT22 cell line after NMDAR knockdown and H/R	↑XLOC_161072, ↑XLOC_065271, ↓XLOC_159404, ↓XLOC_031922	lncRNAs respond to hypoxic stimulation	[147]
Serum of stroke patients	↑HIF1A-AS2; ↓LINK-A	HIF1A-AS2 and LINK-A as diagnostic and prognostic markers	[148]
HIBD neonatal rats	↑151 ↓177	Several lncRNAs can be useful for therapeutic research in neonatal HIBD	[149]

OPC: oligodendrocyte precursor cell; OGD: oxygen-glucose deprivation; ↑: increase; ↓: decrease; lncRNAs: long non-coding RNAs; WMI: white matter injury; bEnd.3: brain microvascular endothelial cells; Gpr137b-ps: G protein-coupled receptor 137b-pseudogene; I/R: ischemic/reperfusion; H/R: hypoxia/reoxygenation; and HIBD: hypoxic ischemic brain damage.

**Table 6:** This table summarizes the studies that reported the effects of circRNA modulation in anoxic/hypoxic conditions.

Models	circRNA Involved	Compound	Results	Ref.
Two RNA-seq datasets (GSE164727 and GSE121178)	2432	-	circRNAs may play a role in HIE development through circRNA/miRNA interactions	[150]
Rats with the right common carotid artery permanently double ligated and sliced from the middle	↑20 ↓46	-	Potential biomarkers or novel therapeutic targets in the treatment of HIBD	[151]
10 peripheral blood samples (6 mL) from neonates with HIE	↑250 ↓206	-	Potential relationship between circRNAs and HIE in neonates	[152]
5 MMD patients	↑54 ↓69	-	Moreover, this study suggest that angiogenesis was involved in asymptomatic MMD	[153]
8 patients harboring saccular UIAs with HR-VWI and 5 controls	412 differentially expressed; RNA hsa_circ_0007990	-	Biomarkers for detecting blood inflammation in people with UIAs accompanied by an AWE	[154]
OGD/R-induced A172 cells tMCAO model	CircFOXP1	-	circFOXP1 reduced apoptosis by affecting Bax and caspase-3 expression.	[155]
BMECs OGD/R-induced Rats with proximal end of the external carotid artery ligated	CircARF3	-	Brain hypoxia-induced damage reduction via miR-31-5p/MyD88/NF-κB axis	[156]
OGD/R-induced HBMECs	Circ0090002	-	Brain hypoxia-induced damage reduction via circ_0090002/miR-186-5p/HECTD1	[157]
Blood samples collected from 25 IS patients and 25 healthy controls+ HBMVECs	Memo1	-	Circ-Memo1 silencing promotes cell viability, inhibiting the activation of ERK/NF-κB signaling pathway; reducing oxidative stress and inflammatory response; and inhibiting cell apoptosis	[158]
OGD/R-induced HBMECs	CircPHKA2	-	circPHKA2 protects HBMEC from OGD-induced neurovascular injuries via circPHKA2/miR-574-5p/SOD2 axis	[159]
OGD/R-induced HBMECs	CircANRIL	-	Knockdown of circRNA_ANRIL improves OGD/R-induced cell damage, apoptosis, and inflammatory responses via circRNA /miR-622/NF-κB	[160]
OGD-induced HBMECs peripheral blood of IS patients	CircFUNDC1	-	CircFUNDC1 knockdown alleviates OGD-induced HBEMCs injuries by targeting the miR-375/PTEN axis.	[161]
NSCs of rat treated with OGD/R	cZNF292	-	cZNF292 silencing alleviated OGD/R-induced injury through the upregulation of miR-22	[162]
H/R-treated HBMECs; MCAO rats	CircDLGAP4	-	Potential key factor in CI/RI treatment	[163]
Mice with high-altitude hypoxia-induced brain injury	23 differentially expressed	*Gymnadenia conopsea* (L.) R. Br.	Potential biomarkers or targets	[165]
HT-22 cells H/R-induced	CircCDR1as	DEX	Circ-CDR1as is downregulated after DEX treatment	[167]

CircRNAs: circular RNAs; HIE:hypoxic ischemic encephalopathy; ↑:increase; ↓: decrease; HIBD: hypoxic ischemic brain damage; MMD: Moyamoya disease; UIAs: unruptured intracranial aneurysms; HR-VWI: high-resolution vessel wall imaging; AWE: abnormal wall enhancement; tMCAO: transient middle cerebral artery occlusion; BMECs: brain microvascular endothelial cells; OGD/R: oxygen-glucose deprivation/reperfusion; HBMECs: human brain microvascular endothelial cells; HBMVECs: human brain microvascular endothelial cells; IS: ischemic stroke; NSCs: neural stem cells; H/R: hypoxia/reoxygenation; CI/RI: cerebral ischemia/reperfusion injury; and DEX: dexmedetomidine.

## Data Availability

Not applicable.
